# An environmental scan of emergency response systems and services in remote First Nations communities in Northern Ontario

**DOI:** 10.1080/22423982.2017.1320208

**Published:** 2017-05-11

**Authors:** E. J. Mew, S. D. Ritchie, D. VanderBurgh, J. L. Beardy, J. Gordon, M. Fortune, S. Mamakwa, A. M. Orkin

**Affiliations:** ^a^ Dalla Lana School of Public Health, University of Toronto, Toronto, Canada; ^b^ School of Human Kinetics, Sudbury, Canada; ^c^ Centre for Rural and Northern Health Research, Laurentian University, Sudbury, Canada; ^d^ Division of Clinical Sciences, Northern Ontario School of Medicine, Thunder Bay, Canada; ^e^ Nishnawbe Aski Nation, Thunder Bay, Canada; ^f^ Northern Ontario School of Medicine, Thunder Bay, Canada; ^g^ Sioux Lookout First Nations Health Authority, Sioux Lookout, Canada; ^h^ Michael G. DeGroote School of Medicine, McMaster University, Hamilton, Canada; ^i^ Schwartz/Reisman Emergency Medicine Institute, Sinai Health System, Toronto, Canada

**Keywords:** Indigenous health, aboriginal health, emergency medical services, remote health, health services, Nishnawbe Aski Nation, environmental scan, community-based participatory research

## Abstract

**Background**: Approximately 24,000 Ontarians live in remote Indigenous communities with no road access. These communities are a subset of Nishnawbe Aski Nation (NAN), a political grouping of 49 First Nations communities in Northern Ontario, Canada. Limited information is available regarding the status of emergency care in these communities.

**Objective**: We aimed to understand emergency response systems, services, and training in remote NAN communities.

**Design**: We used an environmental scan approach to compile information from multiple sources including community-based participatory research. This included the analysis of data collected from key informant interviews (n=10) with First Nations community health leaders and a multi-stakeholder roundtable meeting (n=33) in October 2013.

**Results**: Qualitative analysis of the interview data revealed four issues related to emergency response systems and training: (1) inequity in response capacity and services, (2) lack of formalised dispatch systems, (3) turnover and burnout in volunteer emergency services, and (4) challenges related to first aid training. Roundtable stakeholders supported the development of a community-based emergency care system to address gaps.

**Conclusions**: Existing first response, paramedical, and ambulance service models do not meet the unique geographical, epidemiological and cultural needs in most NAN communities. Sustainable, context-appropriate, and culturally relevant emergency care systems are needed.

## Introduction

In 2015, Canada’s Auditor General identified inequitable health services among remote First Nations communities, including severely under-equipped nursing stations and healthcare staff working beyond their scope of practice [1]. Emergency medical services are among the most deficient. The burden of emergency health conditions among remote First Nations is dramatically elevated compared with other Canadian communities. Elevated rates of chronic and infectious disease manifest as critical health emergencies including mental health and addictions crises, myocardial infarctions, diabetic emergencies, and acute sepsis [1,2]. These service deficiencies coupled with increased risk of emergency health conditions exacerbate the potential for severe illness or death.

## Background

**Figure 1.**
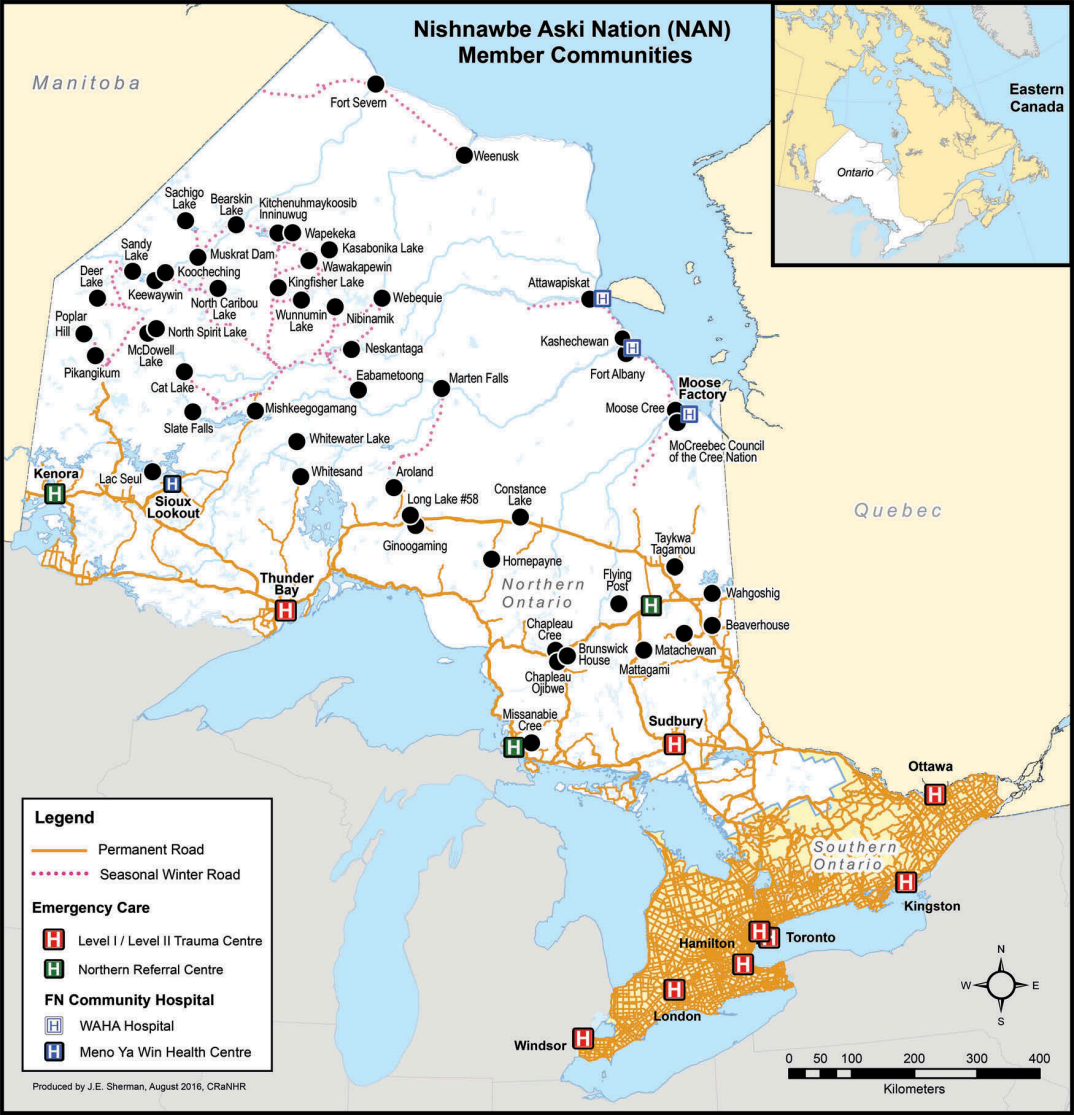
Map of Nishnawbe Aski Nation member communities, road access and nearest emergency care services. (Reproduced with permission from Jill E. Sherman).

The February 2016 Health and Public Health Emergency declaration issued by Nishnawbe Aski Nation (NAN) and the Sioux Lookout Area Chiefs Committee on Health aimed to bring public attention to the healthcare inequities that exist in the province of Ontario within the remote reserves north of Sioux Lookout and NAN Territory [3]. NAN is a First Nations political organisation that represents 49 First Nations communities in northwestern Ontario, many of which are located in remote regions in the far north of the province. Figure 1 geographically depicts NAN communities connected by seasonal and permanent roads, and with their proximity to closest hospitals. The majority of communities are grouped into seven Tribal Councils according to region [4]. Of the estimated 49,000 people represented by NAN on and off reserve, roughly 24,000 live in communities without permanent road access and rely on federal nursing stations or clinics as their only source of local healthcare.

Emergency department care is accessible only by plane or helicopter [1,5]. Air transportation services are operated under air ambulance and private aviation services such as Ornge, the largest supplier, or SkyCare. Landing strips on First Nations reserves often lack navigational equipment available at other airports across Canada [6–9]. Because many of these remote airports are not serviced by instrument approach, pilots must fly “by sight”, limiting the ability to land and take off in adverse weather [8]. Emergency medical evacuations can be delayed as a result of these equipment limitations. These evacuations are not infrequent. Citizens within the remote reserves north of Sioux Lookout face rates of injury five to eight times the national average, accounting for 30% of all deaths in the region [10]. Between January 2012 and September 2013, Ornge performed at least 2033 medical evacuations, equating to 1 medical evacuation for every 12 people over this 21-month period [11].

These communities often do not have formal ambulance services. Despite this disparity in emergency health services, our team was unable to locate scholarly work that systematically described the state of emergency care systems and services in remote NAN communities. The purpose of this study was to better understand the available first response emergency care services and systems in remote NAN First Nations primarily from the beliefs and perspective of community health leaders. Rather than relying solely on government assertions of existing regional or national service provision, we were interested in local perceptions of available care. We defined emergency care as any service that evaluates and/or treats critical medical emergencies to those who are ill and injured, including mental health emergencies. We were guided in our approach by principles of community-based participatory research under a transformative-emancipatory paradigm [12], which posits that the purpose of this project is to improve societal equity for First Nations and addresses power and privilege during the entire research process [13]. We used an environmental scanning (ES) approach to consolidate information from community leaders, frontline practitioners, and publically available sources [14,15]. The first phase of our ES employed an *active approach* by gathering information from primary sources such as perspectives of First Nations health leaders and relevant stakeholders [15]. The second phase employed a *passive approach* by gathering information from secondary sources, such as publically available information, to supplement the results obtained from the active approach [15]. This paper provides the only systematic account of local beliefs and perceptions on the status of emergency response capacity, systems, and training in NAN communities.

## Methods

### Phase I: primary sources

The active approach of the ES consisted of two parts conducted sequentially (1): structured telephone interviews with a group of community health leaders from NAN; and (2) a roundtable with an interdisciplinary group of stakeholders relevant to emergency services in NAN communities. Both the interviews and roundtable process constituted an active approach because the research team interacted with the people and organisations involved in emergency care to both collect data and take action [15]. Both components received ethical approval from Lakehead (#128 12–13/ROMEO 1463141) and Laurentian (#2013–02-11) Universities.

Structured interview questionnaires were developed to assess the status of emergency care within the member communities of each tribal council (Table 1). Ten key informants from the NAN Health Advisory Group agreed to participate in telephone interviews. These informants were invited to participate because they had the collective knowledge of the emergency response systems across NAN communities. Only two of 49 NAN communities were not represented by these informants. Nine informants were community health leaders and one informant was a representative from a local emergency response organisation. Questionnaires were sent to informants prior to each interview. Three team members conducted semi-structured interviews and follow-up phone calls between August and November 2013 using telephone scripts and a data collection template based on the question guide in Table 1. We member-checked our findings by sharing the interview notes with participants and reviewing their responses during follow-up phone calls to confirm accuracy.

**Table 1.** Sample of informant interview questions from telephone scripts.

1. Who responds to health emergencies, like heart attacks, accidental injuries or mental health emergencies, and provides first aid services in the communities you represent? 2. Who trains people to provide emergency first aid in each of the communities you represent? 3. How satisfied are the people of this community/these communities with their emergency response system? 4. Have first response services or training changed in any of your communities in the past 10 years? If yes, can you describe how? 5. Considering the funding, travel, and human resource constraints, what do you believe are the essential elements of an effective emergency response system for a remote NAN community? 6. Do you have anything else you would like to share about pre-nursing station emergency care in the communities you represent?

Quantitative descriptive analyses were conducted using Microsoft Excel. Question-specific response rates (QSRR) differed substantially between questions, as key informants did not always have access to or know relevant information about a particular community they were responsible for. Qualitative content analyses were conducted using NVivo 9.0 to identify: (1) issues related to emergency response systems and training in NAN communities; and (2) essential elements of an effective emergency response system for a remote NAN community. Provisional themes were reviewed and revised by the research team for each analysis to increase reliability [16].

Our team held a two-day multi-jurisdictional meeting in October 2013 with a range of health stakeholders in Sioux Lookout, Ontario (Table 2). The meeting was convened to confirm and interpret the interview data, and to collaboratively develop solutions to improve emergency service limitations and gaps. This included the development of a vision, key recommendations, and guiding principles to improve emergency care services in NAN communities.

**Table 2.** List of representative organisations who attended the multi-jurisdictional roundtable.

Dignitas International Health Canada Independent First Nations Alliance James Bay Ambulance Services (Weeneebayko Area Health Authority) Keewaytinook Okimakanak Matawa First Nations Mushkegowuk Council Nishnawbe Aski Nation Northern Ontario School of Medicine Ontario Ministry of Aboriginal Affairs Ontario Ministry of Health and Long-Term Care Ornge Sachigo Lake First Nation Sandy Lake First Nation Shibogama First Nations Council Sioux Lookout First Nations Health Authority Sioux Lookout Meno Ya Win Health Centre Sioux Lookout Regional Physician Services Incorporated Windigo First Nations Council

### Phase II: secondary sources

The passive approach of the ES consisted of two parts conducted sequentially: (1) reviewing the literature related to emergency response services in NAN communities; and (2) targeted Internet searches of known services to provide information not previously identified. The purpose of this phase was simply to supplement the results of Phase I to identify any additional programmes or services that were previously not mentioned or identified in Phase I. Phase II did not require ethical approval.

We conducted a literature review in February 2016 to identify published and grey literature. The following electronic bibliographic databases were searched: ProQuest, Web of Science, MEDLINE, and Scopus. Inclusion criteria included: (1) NAN community context or authorship; and (2) content primarily related to the delivery and/or access to emergency response services. Citations involving environmental emergency preparedness initiatives were excluded. This search was further supplemented with two independent Google searches including: *“nishnawbe aski nation” emergency services* and *“first nations” emergency services remote ontario*. EM retrieved and reviewed all websites and citations sequentially until saturation. This review also included documents acquired from correspondence between the research team and stakeholders who attended the roundtable meeting.

From the key informant interviews, 12 programmes and services were identified that aimed to provide some degree of emergency care in NAN communities. Targeted website searches were completed in July 2016 to identify publicly available information regarding these 12 programmes.

## Results

### Phase I: primary sources

Quotations are directly from informant interviews.

#### NAN health leader interviews

The majority of NAN communities have limited emergency response resources and services. Although there were ambulance or medical transportation vehicles in 35 (78%) communities (92% QSRR), the condition of vehicles was often unknown and some were believed to be in poor or unusable mechanical condition. Many of these vehicles are regular passenger vehicles not designed for patient transport and medical services, and therefore may be inadequate for the intended role despite being in serviceable condition. It was also outside the scope of practice for Health Canada nurses to respond to emergencies outside of a nursing station, as stated by one informant: “Nurses are now stuck in the nursing station because of the memo.” Although some nurses may not act according to this memo, the policy has nevertheless led to challenges, as another informant indicated that “a patient died on the road in front of the nursing station, and during this incident the nurse could not leave the nursing station”. Despite a lack of permanent road access, the James Bay Ambulance Service operates five paramedic bases in five remote communities in northeastern Ontario: Attawapiskat, Fort Albany, Kashechewan, Moose Factory, and Moosonee. There are no paramedical services in 28 (61%) remote NAN communities (98% QSRR).

Informants identified 12 local programmes, training organisations, health service providers, and political organisations that are involved in emergency care training and response (Table 3). Even when formal emergency response services were available, informants identified a strong community response in attempts to fill service gaps: “there is a reliance on the community to respond to emergencies.” Lay community members such as friends, family and chief and council were known to routinely transport patients to the nursing station in response to all types of emergencies. In communities without access to an ambulance, ill or injured community members are often left to “find their own transportation” to care at the nursing station.

**Table 3.** List of local programs, training organisations, health service providers, and political organisations involved in emergency care training and response.

Canadian Ranger Program Crisis Care Coordinators/Crisis Care Teams operated jointly by NAN and Sioux Lookout First Nations Health Authority Ontario Provincial Ministry of Health & Long Term Care First Nations Emergency First Response Team Program James Bay Ambulance Service Paramedic Program Junior Ranger Program/Ontario Ranger Program Medical Driver Program Nishnawbe-Aski Police Service Ornge/SkyCare Red Cross Sachigo Lake Wilderness Emergency Response Educational Initiative St. John’s Ambulance Service Volunteer Firefighters

The operation of formal programmes is fragmented and heterogeneous. Thirty (79%) communities reported having crisis response teams (78% QSRR) who respond to situations involving suicide attempts and family violence [17]. Only 18 (50%) of communities reported that Canadian Rangers in their community respond to emergencies (73% QSRR) and 18 (39%) of communities reported that paramedical services respond to emergencies (94% QSRR). Communities reported that the provincial Emergency First Response Team (EFRT) programme was active in six (13%) communities (94% QSRR). This programme recruits volunteers from the community to be trained in emergency first response, first aid, and cardiopulmonary resuscitation (CPR) based on provincial first responder standards.

There is limited first aid and CPR training in NAN communities. Three (7%) communities had no first aid or CPR training; the remaining 43 (93%) communities had at least one first aid and CPR training session (98% QSRR). St. John’s Ambulance provided training in 31 (78%) communities (85% QSRR), Canadian Red Cross in 12 (39%) communities (66% QSRR), and the Ontario Ministry of Health and Long Term Care in seven (16%) communities (91% QSRR). Ornge did not provide training in any communities (79% QSRR). Eight (17%) communities had first aid and CPR training from at least two sources, of which six (75%) received services from St. John’s Ambulance and the Red Cross. Other sources of training cited by informants included: the Aboriginal Health and Wellness Strategy, the Canadian Ranger Program, James Bay Emergency Medical Services, and the Sachigo Lake Wilderness Emergency Response Educational Initiative. First aid and CPR training frequency ranged from “twice per year” to every “three to four years”. Estimated training frequency averaged once every 2 years (81% QSRR); although many comments indicated that training frequency varied significantly or was irregular with many years between training cycles. Average length of training was 2.7 days (87% QSRR) ranging from “two to three days” to “five days plus two-day training in Sioux Lookout”. An estimated average number of 18 people per community were trained with each training session, ranging from two to 200 people. Forty-two (91%) communities identified that trainers were from outside the communities (98% QSRR).

Four themes emerged related to the issues surrounding emergency response systems and training in NAN communities (Table 4):

1. *Inconsistent and inadequate response capacity and services*. There is a wide variety of emergency response capacity and services across NAN communities, as “different communities have different emergency services available to them”. Some communities have access to paramedic services, such as five communities serviced by James Bay Ambulance Service, or have road access and are serviced by the existing provincial system; however, many smaller remote communities have limited, if any, response capacity and services: “the community doesn’t have a program to address emergency response [nor]…an ambulance response service, and that is required. They make use of what they have. They’ve been lucky so far.”
2. *No formalised emergency service dispatch system*. There is a lack of consistent, reliable and standardised communication. Many communities lack 911 services, cell services, reliable landlines, and street names or household identifiers. Often the communication system is unclear, requiring two or three different phone calls during times of emergency: “when there is an issue or emergency in a community, community members call at least two or three different numbers. At times, there is no 24 hours per day coverage, so the calls go unanswered, and it is then the responsibility of the community members to get the sick or injured person to the nursing station.”
3. *Turnover and burnout in volunteer teams*. Some communities have volunteer-run EFRTs that support pre-nursing station care; however, there is high volunteer turnover where the team is dispatched often: “being called repeatedly at 3 am, 4 am, or 5 am is draining for volunteers, and if there is too much demand, the team turns over frequently.”
4. *Challenges related to first aid training for community members*. There is a wide variety in first aid training, providers, and curriculum. Urban-developed first aid training courses are not appropriate in communities without paramedical services. For example, “patient transfer is not covered in normal CPR and first aid training, which is a problem … [because] training usually assumes that there are ambulance and 911 services nearby”. Training in many remote communities is infrequent, inconsistent, or does not occur at all. Training is usually only 2–3 days long and is often only provided to staff, employees, or teams in a community: “there is no regular [first aid] training”, “first aid training is way too short”, and “trainers are from outside the communities [and] this is part of what makes it challenging”.

**Table 4.** Themes extracted from key informant interviews identifying challenges within existing emergency response system in NAN communities and components of a perceived effective emergency response system for remote NAN communities.

Issues in existing emergency response systems	Components of an effective emergency response system
● Inconsistent and inadequate response capacity and services.	● Reliable emergency service dispatch system.
● No formalised emergency service dispatch system.	● Support for volunteer emergency response teams.
● Turnover and burnout in volunteer teams.	● Reliable and responsive transportation.
● Challenges related to first aid training for community members.	● Context-relevant system infrastructure.
	● Training that is reliable and context appropriate.

Five themes emerged related to identifying the essential elements of an effective emergency response system for a remote NAN community (Table 4):

1. *Emergency service dispatch system*. Community members need access to an effective system of communication to access first responders in an emergency: “A 911 for the North is needed.” Since people frequently travel in, out, and between remote communities, one respondent indicated that “a standardised system is required that is consistent across communities”. Another critical element of an effective emergency dispatch system is reliability: “When there is an issue or emergency in a community, the members call at least two or three different numbers (nursing station, on-call person, Nishnawbe-Aski Police Service). At times there is not 24 hour per day coverage, so the calls go unanswered, so it is then the responsibility of the community members to get the sick/injured person to the nursing station.”
2. *Support for volunteers*. Existing volunteer emergency response teams need to be trained, equipped, and supported to enhance continuity to prevent burnout and turnover: the life expectancy of a team member is directly proportional to the call volume”, and “turn-over is problematic since the new people start at square one again.” One respondent summarised the challenge facing the use of EFRT programme: Most people dedicate themselves to the training and find the long-term commitment far too extensive, or find this is something they pursue as a career and leave to acquire the education needed to become a member of the medical field. In our region, emergency response teams will have cycled out a complete membership after 2 years with the exception usually of one or two people who are truly committed and suited to emergency work.”In summary, “The proper support and coordination of a team is required to ensure any measure of longevity”. Volunteer emergency care positions must be supported by paid professional or paraprofessional roles.
3. *Reliable and responsive transportation*. There needs to be a safe, reliable and timely transportation system for patient preparation and transition to the nursing station or health centre. “Each community should have an emergency transport vehicle or ambulance.” In addition, the transport system may need to include “boat, motor, skidoo and sled for winter”. Considering the geographical context of large tracts of wilderness surrounding remote communities, one respondent summed this need succinctly: “safe transfer to a vehicle and safe transport to the nursing station.”
4. *Context-relevant system infrastructure*. The emergency response system needs to be adapted and standardised for small remote communities, and this requires infrastructure funding and support for resources, training, and equipment. One respondent stated: “Each community should be outfitted with the resources (equipment and people) necessary to be able to respond to emergencies in the community.” Patient transition to the nursing station and health centres needs to reflect the reality of the community context with strong working relationships between nurses and first responders: “There should be a strong working relationship and system in place with the nursing station so the transition occurs smoothly and effectively.”
5. *Training that is reliable and context appropriate*. Training needs to be longer than 3 days, more frequent, and more consistent: “There needs to be consistency in trained people that are able to respond to emergencies” and “regular refresher courses in the communities as a preventative measure, perhaps once a year to keep people fresh and up-to-date”. The curriculum needs to be adapted to deal with relevant critical health emergencies and adjusted to non-urban contexts without typical emergency response systems: “First aid & CPR deal with physical health, but communities need mental health first aid as well to deal with abusive situations, suicide and situations involving drugs and alcohol.” Community members should be encouraged to participate in training because they are a valuable first response resource in each community as “training is key and this includes training for lay community members”.

#### Multi-jurisdictional roundtable

Representatives of this roundtable included an interdisciplinary group of 33 partners including 16 (48%) representatives from First Nations governance and community organisations, seven (21%) representatives from Ontario Provincial and Canadian Federal governments, seven (21%) representatives from nursing and paramedical services, and three (9%) representatives from non-governmental organisations.

Representatives confirmed the accuracy of results from the informant interviews and interpreted these results to identify improvement opportunities for NAN communities. There were two main outcomes from this meeting: (1) a shared vision for the future of emergency medical services in NAN communities; (2) guiding principles central for implementation of the vision. The shared vision is: “people in remote and isolated First Nation communities should have access to excellent community-based first response emergency care” [5]. The six guiding principles for advancing solutions in pre-nursing station care in remote NAN communities are: community-based, sustainable, capacity-building, collaboration, integration, and excellence (Table 5). The roundtable meeting also led to the identification of two recommended actions including: (1) collaboration between NAN and Federal and Provincial governments; and (2) plan and test a community-based approach to emergency care in partnership with a selection of NAN communities. This approach has been described elsewhere [18]. The meeting report “Community-Based Emergency Care: An Open Report for Nishnawbe Aski Nation” is a public summary of this meeting and it is available at www.nosm/cbec [5].

**Table 5.** Summary of guiding principles developed by stakeholders at the multi-jurisdictional roundtable meeting to guide building an effective emergency response system in remote NAN communities.

Guiding Principle	Description
Community-based	Identify, respect, and learn from the diversity of remote and isolated First Nation communities. Address individual and population health needs by building on local priorities, relationships, skills, strengths and culture. Develop, deliver and evaluate programs with the community, and for the community.
Sustainable	Strive for lasting and scalable community-based emergency care programmes, rooted in sound health, human resources, economic, and community planning. Build on opportunities to develop community resilience and health services as a sustainable and renewable local resource.
Capacity building	Build capacity by providing emergency care training across a large cross-section of community members. Explore opportunities to develop employment opportunities for local emergency care and training.
Collaboration	Work with partners in healthcare delivery, such as community health workers, nurses, paramedics and physicians in the design, delivery, evaluation and funding of community-based emergency care programmes. Develop programmes as a collaboration between First Nations and local, provincial and federal governance organisations.
Integration	Ensure that community-based emergency care programmes integrate with emergency health services provided by nurses, paramedics and physicians, as well as other community emergency management strategies including Canadian Rangers and Crisis Response Teams.
Excellence	Evaluate and study programmes in collaboration with communities, to bring high-quality, equitable, innovative and evidence-based emergency care to ill and injured patients in remote communities.

### Phase II: secondary sources

The literature search yielded 53 citations, four of which met inclusion criteria. Two news articles stated the need for improved emergency response from First Nations leaders [19,20]. The remaining two articles were research publications describing the Sachigo Lake Wilderness Emergency Response Educational Initiative [21,22]. Google searches yielded 31 relevant websites. Two sources were statements from First Nations leaders calling for improved pre-nursing station emergency response services [20,23]. The remaining websites did not provide novel information.

We could not locate significant amounts of publicly available information for the majority of programmes and services. Those that could be located provided no novel information. Documents from professional correspondence revealed that the Canadian Red Cross developed a partnership with Moose Cree First Nation in 2012 called the *Strength & Spirit Campaign* to develop and deliver existing and novel Red Cross programmes and services to better meet community needs [24].

## Discussion

Existing emergency response systems, efforts, and programmes are inadequate in NAN communities. Given the small population sizes and a variety of other contextual, historical, and geographical factors, many communities do not have 911 services or other essential emergency prevention and response systems. There are several programmes and services that address pre-nursing station care; however, the operation of these programmes is heterogeneous and fragmented. Many existing programmes depend on volunteers for operation, leading to burnout, turnover, and unreliable care. These challenges often place the burden on community members to transport patients to the nursing station; however, there is also lack of equitable, frequent, and effective training opportunities to ensure that lay responders are adequately prepared to manage the medical emergencies that occur in rural and remote NAN communities.

Themes related to issues in emergency care that were identified from interviews with NAN health leaders included limited capacity, services, formalised dispatch systems, and contextually appropriate models of emergency medical service delivery. The expansion of conventional ambulance, first responder, and first aid programmes in these isolated and remote First Nations may not meet the unique geographical, epidemiological, and cultural needs of communities [18]. Standard first aid protocols and equipment in such settings with limited resources, isolated contexts, and minimally trained providers will not ensure quality care [18]. In some cases, basic first aid standards may even be ineffective [18].

Innovative, sustainable, and community-based innovations in emergency health services delivery are urgently needed. Local First Nations emergency response stakeholders agreed that “people in remote and isolated communities should have access to excellent community-based first response emergency care”. Possible benefits for a community-based emergency care model include reduction in morbidity and mortality, the building of safer and more resilient communities, more local health knowledge and leadership, enhanced emergency response and crisis management capacity, enhanced community ownership and self-determination of health services, and economic development for remote communities [5]. In the absence of local paramedical services, the management of health emergencies depends on the capacity of lay community members. The addition of a comprehensive community-based pre-nursing station care model could reduce morbidity and mortality.

There are limitations to this study. Most of the data were based on interview responses and were not subject to verification from other community experts. Informants were responsible for up to eight First Nations communities, and this increased the challenge of accurately recalling or describing local contexts; however, many informants did take the time to research their answers. This increased the overall accuracy of the results reported, and therefore the results most likely provide a reliable assessment of local services and resources. Individual quotations may not be representative of overall trends or perceptions, but given that our respondents were legitimate representatives of a regional First Nations governance organisation, we feel that they represent credible perspectives. The passive phase of the project was conducted nearly 3 years after the active approach, so local circumstances may have changed in the intervening years; however, secondary sources did not identify any evidence that there has been improvement since the time of the interviews in 2013.

Emergency response systems, efforts, and programmes are grossly insufficient in NAN communities. As these communities operate in isolated and remote contexts, the expansion of conventional ambulance or first responder programmes may not be an appropriate nor realistic solution. Remote communities are the most vulnerable, and transformation in health care to improve equity is needed. It is therefore paramount that novel, sustainable and community-based innovations are developed, particularly those addressing pre-nursing station care. The results of this paper can empower NAN communities with the information to advocate for improved emergency services and systems, which will then result in healthier, stronger, and more resilient communities.
